# Low Oxygen Enhances Primitive and Definitive Neural Stem Cell Colony Formation by Inhibiting Distinct Cell Death Pathways

**DOI:** 10.1002/stem.96

**Published:** 2009-08

**Authors:** Laura Clarke, Derek van der Kooy

**Affiliations:** Institute of Medical Science, University of TorontoToronto, Ontario, Canada

**Keywords:** Neural stem cell, Apoptosis, Hypoxia, Embryonic stem cell, Caspase

## Abstract

Neural stem cells (NSCs) can be derived from single mouse embryonic stem cells (ESCs) in the absence of instructive factors. Clonal primitive NSC (pNSC) colonies are formed first, and then give rise to clonal, fibroblast growth factor-dependent definitive neural stem cells (dNSCs). We tested low-oxygen culture as a potential method of alleviating the extensive cell death seen in pNSCs and dNSCs. Culture in low (4%) oxygen promoted survival of pNSCs by inhibiting apoptosis-inducing factor (AIF)-dependent cell death, although pNSCs undergo both AIF- and caspase-mediated cell death in 20% oxygen. In contrast, survival of dNSCs in low oxygen was increased by inhibition of caspase-dependent cell death. In normoxia, AIF is implicated in promoting dNSC survival. Neither survival effect was dependent on the main transcriptional effector of hypoxia, hypoxia-inducible factor 1. Low-oxygen concentrations may be involved in expansion of early NSC populations by inhibiting cell death through different pathways in these sequential pNSC and dNSC populations. Stem Cells 2009;27:1879–1886

## INTRODUCTION

Neural stem cells (NSCs) are present in the mouse embryo early in development. The first NSCs to arise are primitive neural stem cells (pNSCs), which can be isolated from the anterior embryo from embryonic day (E)5.5 epiblast until E8.5 neuroectoderm. The properties of pNSCs are intermediate between embryonic stem cells (ESCs) and definitive neural stem cells (dNSCs). pNSCs transition to dNSCs in the embryo at approximately E8.5 and dNSCs are present in the brain throughout the life of the organism [1].

This NSC lineage can be modeled in vitro by placing single ESCs in minimal media, where cells rapidly adopt a neural fate in the absence of instructive factors. pNSCs derived from ESCs in this colony-forming assay upregulate expression of neural markers such as *nestin* and *sox1* while downregulating genes associated with mesoderm, endoderm, and epidermis [2,3]. Like ESCs, pNSCs proliferate in response to leukemia inhibitory factor (LIF) and maintain expression of *oct4*. pNSCs can be passaged to give rise to dNSC colonies dependent on fibroblast growth factor (FGF). dNSC colonies can then be passaged indefinitely. A challenge in culturing pNSCs is the extensive cell death that occurs when ESCs are placed into minimal, serum-free conditions. Genetic manipulation of cell death pathways has shown that pNSCs undergo both caspase-dependent and apoptosis-inducing factor (AIF)-dependent cell death, and the addition of survival factors in the colony-forming assay increases the efficiency of deriving pNSC colonies from ESCs 100-fold [3].

Another potential method of alleviating cell death in ESC-derived NSCs is the use of low-oxygen conditions, as the culture of stem and progenitor cells in low oxygen has positive effects on survival and proliferation in many systems [4]. Oxygen levels found in body tissue are much lower than the 20% oxygen present in standard culture conditions. It has been proposed that some tissue-specific stem cells exist in a low-oxygen niche [5], thus exposure to ambient room oxygen levels may be particularly harmful to these populations. Genes regulated by oxygen include those involved in glycolysis, angiogenesis, and hematopoiesis, and so oxygen levels also may alter fate determination. Low-oxygen culture has been reported to affect the differentiation of brain-derived NSCs [6,7].

In this study we investigated the effect of low (4%)-oxygen culture on ESC-derived NSCs. We hypothesized that culture in relative hypoxia would be an effective means of attenuating the cell death that occurs when ESCs are placed into the neural colony-forming assay. Low-oxygen culture also more closely resembles the in vivo environment of pNSCs and dNSCs, particularly as they arise in the embryo before a functional circulatory system [1]. Thus, we also tested whether low-oxygen culture influences the in vitro acquisition of a neural fate.

## MATERIALS AND METHODS

### Low-Oxygen Culture

In the normoxic condition, cells were placed in a 37°C incubator supplemented with 5% CO_2_. For low-oxygen culture, 4% oxygen, 5% carbon dioxide, and nitrogen gas were mixed using compressed air and supplied to a sealed container with a small outtake valve placed inside a 37°C incubator.

### ESC Culture

ESCs were maintained on mitotically inactivated mouse embryonic fibroblasts in Dulbecco's modified Eagle's medium containing 15% fetal calf serum (FCS) and 1000 U/ml LIF. R1 ESCs were used unless otherwise noted. Other ESC lines used were *hif1*α^−/−^ (a gift from Peter Carmeliet) [8], *flk*^−/−^ [9], *RBPJ*κ^−/−^ [10], E14K, *aif-/Y* [11], and *caspase9*^−/−^ [12].

### Neural Colony-Forming Assay

The colony-forming assay was carried out as previously described [2,3]. Briefly, ESCs were harvested, washed twice in serum-free medium, and plated as single cells in serum-free medium containing LIF (1000 U/ml) in 24-well plates (Nunclon; Nunc, Rochester, NY, http://www.nuncbrand.com) at a density of 10 cells/μL or less. Over a period of 7 days, floating sphere colonies formed. pNSC colonies were passaged to dNSC colonies by dissociating pNSC colonies with TrypLE (Invitrogen, Carlsbad, CA, http://www.invitrogen.com) followed by brief manual trituration, and plating at 10 cells/μL or less in serum-free media supplemented with 10 ng/ml FGF2 (Sigma-Aldrich, St. Louis, http://www.sigmaaldrich.com), 2 μg/ml heparin (Sigma-Aldrich), and B27 (Invitrogen). Cells were assessed for viability using trypan blue exclusion immediately before plating. For differentiation, pNSC colonies were dissociated into a single-cell suspension and plated on Matrigel (BD Biosciences, San Diego, http://www.bdbiosciences.com) in serum-free media containing 1% FCS, then processed for immunocytochemistry after 7 days.

### Cell Survival and Proliferation

To assay cell survival, single ESCs were plated in 96-well plates in serum-free media at low density (100 cells/well). At 4, 24, or 48 hours the number of live cells/well was counted using trypan blue exclusion. Proliferation was determined using bromodeoxyuridine (BrdU) incorporation. Fifty or 100 single ESCs were plated in a 96-well plate in serum-free media containing LIF. Twenty-four or 48 hours later, cells were pulsed with 0.6 μM BrdU (Sigma-Aldrich) for 2 hours and then fixed with 4% paraformaldehyde and processed for immunocytochemistry.

### Brain-Derived Neural Stem Cell Culture

NSCs were isolated from the adult subependyma or the E14 ganglionic eminence using a clonal neurosphere assay [13]. For adult brains, the subependymal zone was microdissected and dissociated using trypsin, hyaluronidase, and kynurenic acid (all from Sigma-Aldrich) followed by manual trituration. Embryonic ganglionic eminence was isolated and dissociated by manual trituration. Cell viability was assessed immediately before plating using trypan blue exclusion and adult or embryonic cells were plated at 10 and 5 cells per microliter, respectively, in serum-free media containing 10 ng/ml FGF2, 20 ng/ml epidermal growth factor, and 2 μg/ml heparin (all from Sigma-Aldrich). Colonies were counted after 7 days.

### Immunocytochemistry

Cells were fixed with 4% paraformaldehyde and permeabilized using 0.3% Triton-X in phosphate-buffered saline (PBS) before blocking in 10% normal goat serum (Jackson Immunoresearch Laboratories, West Grove, PA, http://www.jacksonimmuno.com) for 1 hour. Primary antibodies used were anti-betaIII-tubulin (1:500, Sigma-Aldrich), anti-BrdU (1:500, Abcam, Cambridge, U.K., http://www.abcam.com) and anti-activated caspase3 (1:500, Promega, Madison, WI, http://www.promega.com). Secondary goat anti-mouse (1:400, Alexa) and donkey anti-rat (1:250, Jackson) antibodies were used for detection. Nuclei were counterstained with Hoechst. Cells were visualized in PBS at room temperature using a Zeiss Axiovert inverted fluorescence microscope and images were acquired with AxioVision v4.6 imaging software and AxioCam MRm camera with monochrome CCD sensor (all from Carl Zeiss, Jena, Germany, http://www.zeiss.com).

### Flow Cytometry

Cells were stained with propidium iodide (2.5 μg/μL) and sorted using a BDFacsCanto (both from BD Biosciences). Analysis was performed using BD FacsDiva Software (BD Biosciences).

### Quantitative Reverse Transcription PCR

RNA was extracted using a Qiagen RNeasy extraction kit (Hilden, Germany, http://www1.qiagen.com) with DNase to remove genomic DNA contamination. RNA was quantified using Nanodrop and a specified amount of cDNA was reverse transcribed using SuperscriptIII (Invitrogen). Polymerase chain reaction (PCR) was carried out using Taqman Gene Expression Assays for *sox1, nestin, sox2, oct4, nanog*, and *brachyury* in a 7900HT Fast Real-Time PCR System (Applied BioSystems, Foster City, CA, http://www.appliedbiosystems.com). Quantification was performed using the delta C_t_ method with *hprt1* as an endogenous control template as levels were constant between conditions.

### Statistics

Statistical analysis was performed using SigmaStat 3.1. Student's *t* tests and analysis of variance with multiple comparisons using the Holm-Sidak method were used as appropriate with an overall significance level of .05.

## RESULTS

### Low-Oxygen Culture Enhances Colony Formation by Increasing Primitive Neural Stem Cell Survival

To determine the effect of oxygen levels on the derivation of pNSCs from mouse ESCs, we cultured cells in either a standard incubator supplied with 5% CO_2_ and 20% oxygen or a low-oxygen incubator supplied with 5% CO_2_ and 4% oxygen, with nitrogen gas making up the difference.

**Figure 1:**
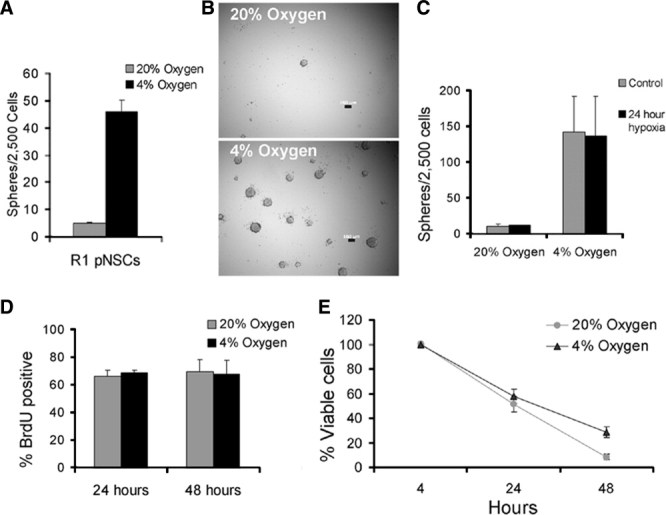
Low oxygen enhances pNSC colony formation by promoting pNSC survival. **(A, B):** The number of pNSC colonies that formed in 4% oxygen culture was approximately 10-fold greater than in control, 20% oxygen culture, t_4_ = 34.9, *p* < .05, *n* = 3. Bar in **(B)** = 100 μm. **(C):** Embryonic stem cells (ESCs) pre-exposed to 4% oxygen before being placed into the default assay did not show a difference in colony number, suggesting that oxygen does not act by driving ESCs toward a pNSC fate, F_1,7_ = 0.007, *p* > .05, *n* = 2. **(D):** Twenty-four hours (t_4_ = 0.63, *p* > .05, *n* = 3) or 48 hours (t_4_ = 0.179, *p* > .05, *n* = 3) after being placed into the neural default assay, ESCs that had transitioned into pNSCs proliferated at similar rates in high and low oxygen, assayed by 2-hour BrdU incorporation. **(E):** Increased pNSC colony formation in the low-oxygen condition is due to improved cell survival. The number of viable cells present in 4% oxygen was significantly higher than in 20% oxygen by 48 hours after being placed into the default assay as measured by trypan blue exclusion assay, F_1,17_ = 11.1, *p* < .05, *n* = 3. All data are mean ± SEM. Abbreviations: BrdU, bromodeoxyuridine; pNSC, primitive neural stem cell.

We found that pNSC colony formation was enhanced dramatically in low-oxygen conditions (Fig. 1A, 1B). The number of colonies that formed in low-oxygen culture was approximately 10-fold greater and colony size was 48.7% ± 3.8% larger than in the normoxic condition. This enhancement could have been due to increased proliferation, increased survival, or a greater number of ESCs defaulting to the neural fate. As greater than 90% of single ESCs plated under these conditions acquire a neural fate [3], the latter possibility is unlikely to account for the increase we observed. ESCs acquire a neural fate within hours after being placed into the colony-forming assay [3]. Thus it was likely that the effect on pNSC colony formation was due to direct effects on pNSCs, and not on ESCs. To exclude the possibility that low-oxygen culture selects for a fraction of ESCs competent to acquire a neural fate, ESCs were transferred to 4% oxygen for 24 hours prior to being placed in the colony-forming assay. Pre-exposure to 4% oxygen had no effect on the number of pNSC colonies that formed at 20% oxygen, or the increased colony number in 4% oxygen (Fig. 1C). To determine whether low oxygen promotes pNSC proliferation, ESCs were plated in the colony-forming assay for 24 or 48 hours under normoxia or low oxygen and then pulsed with the thymidine analogue BrdU for 2 hours. Staining for BrdU revealed no significant increase in the proportion of proliferating cells at either time point (Fig. 1D). This suggested that survival of pNSCs was higher in low-oxygen culture. To test this, single ESCs were plated in serum-free media at 20% and 4% oxygen, and the number of viable cells was counted after 4, 24, and 48 hours. Plates from low-oxygen culture had 3 times more surviving cells 2 days into the assay (Fig. 1E), suggesting that increased survival in low-oxygen culture leads to the increase in pNSC colony number. In addition, analysis of cells by flow cytometry showed that 75.4% ± 4.4% of all cells in the normoxic condition were undergoing cell death as indicated by propidium iodide incorporation after 48 hours in the colony-forming assay, compared with 65.3% ± 2.6% of cells in low oxygen (*n* = 4). Caspase-dependent cell death was found to be significantly more prevalent in normoxia, with 27.6% ± 5.1% of cells staining positive for activated caspase3 at 48 hours, compared with 13.9% ± 1.8% in 4% oxygen (t_6_ = 2.94, *p* < .05)

### Increased pNSC Survival Is Independent of Hypoxia-Inducible Factor 1 and its Targets, But Is Mediated Through an Inhibition of Apoptosis-Inducing Factor

**Figure 2:**
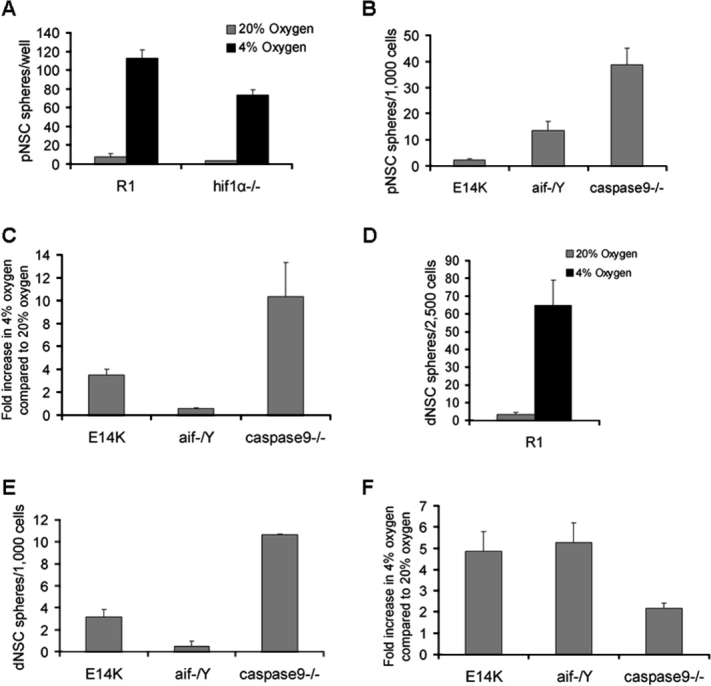
Increased pNSC survival in low-oxygen culture is independent of hypoxia-inducible factor 1 and dependent upon inhibition of apoptosis-inducing factor (AIF)-mediated cell death, whereas increased dNSC survival involves inhibition of caspase9. **(A):** *hif1*α^−/−^ embryonic stem cells (ESCs) showed increased pNSC colony formation in 4% oxygen, similar to wild-type R1 ESCs, F_1,15_ = 303.8, *p* < .05, *n* = 4. **(B):** In the normoxic condition, *caspase9*^−/−^ ESCs showed the most enhanced colony formation over wild-type E14K cells in normoxia, and a smaller increase was seen for *aif-/Y* ESCs, F_2,12_ = 31.9, *p* < .05 for each, *n* = 4. **(C):** E14K and *caspase9*^−/−^ pNSC colony numbers were improved by low oxygen, however increased colony formation in low oxygen was blocked by the absence of AIF, F_2,10_ = 44.0, *p* < .05, *n* = 3. **(D):** There was a near 10-fold increase in dNSC colonies in low-oxygen culture, t_4_ = 8.25, *p* < .05, *n* = 3. **(E):** *Caspase9*^−/−^ pNSC colonies gave rise to more dNSC colonies than the wild-type E14K line. However, very few dNSC colonies arose from *aif-/Y* pNSC colonies, F_2,11_ = 171.8, *p* < .05 versus E14K for each, *n* = 4. **(F):** Both E14K and *aif-/Y* dNSC colony numbers were increased by low-oxygen culture, however *caspase9*^−/−^ dNSCs showed a significantly smaller enhancement in low oxygen, F_2,11_ = 6.26, *p* < .05 versus E14K for *caspase9*^−/−^, *p* > .05 versus E14K for *aif-/Y*, *n* = 4. Data in **(A, B, D, E)** are mean sphere number ± SEM; data in **(C, F)** are expressed as mean fold increases ± SEM in colony number in 4% oxygen over 20% oxygen. Abbreviations: dNSC, definitive neural stem cell; pNSC, primitive neural stem cell.

Adaptive responses to low oxygen in mouse ESCs are mediated at the level of transcription by hypoxia-inducible factor 1 (HIF1) [14,15]. Targets of HIF1 include genes involved in metabolism, as well as in cardiovascular development and angiogenesis. We investigated pathways activated by HIF1 for their involvement in enhanced pNSC colony formation. Vascular endothelial growth factor signaling through the Flk receptor has been demonstrated to influence survival of both pNSCs and dNSCs [16], however, pNSCs derived from ESCs lacking the Flk receptor showed a response to low oxygen (pNSC colonies in 20% oxygen: 7.9 ± 3.0, in 4% oxygen: 79.8 ± 26.1). The Notch pathway is involved in the maintenance of dNSCs, and has been implicated in maintaining NSCs in an undifferentiated state [1,17,18]. We found that pNSCs null for the common downstream effector of Notch, RBP-Jκ, were also strongly affected by low oxygen (pNSC colonies in 20% oxygen: 8.96 ± 1.28, in 4% oxygen: 83.8 ± 10.4). In addition, erythropoietin, demonstrated to influence NSC differentiation [7] did not reproduce the effects of low-oxygen culture when added to the media in the colony-forming assay (not shown). Given that none of these factors was required for enhanced pNSC colony formation, we asked whether HIF1 itself was necessary for this effect. *hif1*α^−/−^ ESCs (a gift from Dr Peter Carmeliet) lack the alpha subunit of HIF1. HIF1α is rapidly degraded in normoxia but not in low oxygen. These cells had a low baseline colony formation, however *hif1*α^−/−^ ESCs placed in the colony-forming assay also showed significantly enhanced pNSC colony formation (Fig. 2A). Although the low numbers of *hif1a*^−/−^ pNSC colonies suggest that it may play a role in this lineage, the effect of low-oxygen culture on pNSCs is independent of HIF1.

We next focused directly on cell death occurring in the colony-forming assay. Previous work [3] has shown that pNSCs undergo both caspase-dependent and caspase-independent programmed cell death. We confirmed this using *caspase9*^−/−^ and apoptosis-inducing factor (*aif*)-/*Y* ESCs to examine caspase-dependent and -independent cell death. In normoxia, both *caspase9*^−/−^ and *aif*-/*Y* ESCs gave rise to more pNSC colonies than wild-type E14K cells (Fig. 2B). Low oxygen could act by attenuating either or both of these cell death pathways. When *caspase9*^−/−^ and *aif-/Y* ESCs were placed into the colony-forming assay at 20% and 4% oxygen, there was a significant effect of genotype on the fold increase in pNSC colony number in low oxygen. *Caspase9*^−/−^ ESCs showed a large increase in pNSC colony formation in low oxygen (Fig. 2C). In *aif-/Y* ESCs, low oxygen did not result in any significant further increase in pNSC colony formation over the 20% oxygen condition. This lack of response of *aif-/Y* mutant pNSCs to 4% oxygen implicates an inhibition of this pathway as the mediator of improved pNSC survival in low-oxygen cultures.

### dNSC Colony Formation Is Increased in Low-Oxygen Culture Partially Through Inhibition of Caspase-Dependent Apoptosis

When pNSC colonies are dissociated into single cells and plated in the presence of FGF and absence of LIF, dNSC colonies arise. dNSC colony formation also was enhanced greatly in low-oxygen culture (Fig. 2D), and this enhancement also occurred in the absence of *hif1*α (not shown). To determine whether the increase in dNSC colony formation in low oxygen was due to the same mechanism as in pNSCs, we derived dNSC colonies from E14K, *aif*-/*Y*, and *caspase9*^−/−^ pNSCs. When plated in normoxia, there was a significant difference in dNSC colony number among genotypes. Whereas the number of pNSCs that formed from *caspase9*^−/−^ ESCs was greater than the wild-type E14K line, fewer dNSCs arose from *aif-/Y* pNSCs, suggesting that dNSCs do not normally undergo AIF-mediated cell death, and that AIF may actually be involved in promoting dNSC survival (Fig. 2E, compare with Fig. 2B). When E14K, *aif*-/*Y*, and *caspase9*^−/−^ dNSCs were cultured in 4% oxygen, there was a significant difference in fold colony increase in 4% oxygen over 20% oxygen among genotypes (Fig. 2F). Although colony number was very low, *aif-/Y* dNSC colony formation was enhanced by low-oxygen conditions similar to wild-type cells. Caspase9 mutant dNSCs showed only a modest increase in colony formation in low oxygen. The effect was smaller than the enhancement seen in wild-type E14K dNSC colonies, suggesting that low oxygen partially acts by decreasing caspase9-mediated cell death in dNSCs. The response to low oxygen in *caspase9*^−/−^ dNSCs was significantly smaller than in *caspase9*^−/−^ pNSCs, supporting the idea that inhibition of caspase-dependent apoptosis is more important for low-oxygen effects on dNSCs than on pNSCs (compare Fig. 2C and Fig. 2F). These results reveal a double dissociation of cell death pathways inhibited by culture in low oxygen. AIF and not caspase9 is required for enhanced pNSC colony formation in low oxygen, whereas caspase9 and not AIF is important for increased dNSC colony numbers in low oxygen.

We used the antioxidant *N*-acetyl cysteine (NAC), an inhibitor of AIF to verify our results in R1 NSCs. We found that pNSC colonies were increased by treatment with 1 mM NAC, and that fewer pNSC colonies formed in 4% oxygen in the presence of NAC compared with in 20% oxygen with NAC (supporting information Fig. 1A, 1B). dNSC colony formation was not inhibited by NAC, but was significantly increased (supporting information Fig. 1C). dNSC colony formation further increased in NAC-treated, low-oxygen condition (supporting information Fig. 1D). This suggests that, in dNSCs, there are distinct, NAC-insensitive, survival-promoting and NAC-sensitive, cell death-promoting roles of AIF. We found that, in our assay, caspase inhibitors were unable to reliably increase colony formation and that cells experienced toxicity due to nonspecific effects.

### Low-Oxygen Culture Does Not Alter the Default Acquisition of Neural Identity

**Figure 3:**
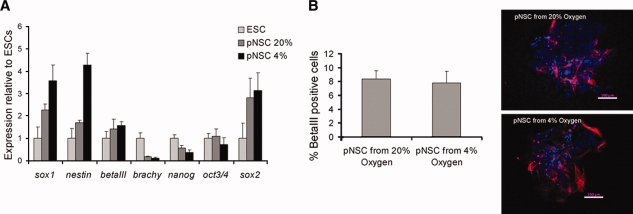
Derivation of pNSCs in 4% oxygen does not alter acquisition of a neural fate. pNSCs grown in 4% oxygen for 7 days upregulate the neural markers *sox1, sox2, nestin*, and *betaIII tubulin* and downregulate the mesoderm marker *brachyury* relative to starting ESC populations. Levels of *oct4* and *nanog* were unchanged between pNSCs in 20% and 4% oxygen, *n* = 4 **(A)**. **(B):** Differentiation of pNSCs grown in high and low oxygen showed that cells from both conditions produced beta-III tubulin-positive neurons at the same frequency, t_4_ = 0.34, *p* > .05, *n* = 3. Bar in **(B)** = 100 μm. Abbreviations: ESCs, embryonic stem cells; pNSC, primitive neural stem cell.

Low-oxygen culture maintains some progenitor cell types in an undifferentiated state [18]. To determine whether culture in 4% oxygen inhibits cells from entering the neural lineage, we performed quantitative PCR analysis of pNSC colonies grown in high and low oxygen. In both conditions, pNSCs showed upregulation of the neural markers *sox1, sox2, nestin*, and *betaII tubulin* relative to ESCs, and downregulation of the mesoderm marker *brachyury*. There was also no increase in expression of pluripotency-associated genes *oct4* and *nanog* in pNSCs from low oxygen, compared with those from high oxygen. (Fig. 3A). We tested the potential of pNSC colonies from low- and high-oxygen culture by dissociating spheres from each condition and allowing them to differentiate in the presence of serum for 1 week. There was no difference in the proportion of cells differentiating into neurons between the two conditions (Fig. 3B), and the small number of glial cells produced was also unchanged (not shown).

### dNSCs from Adult or Embryonic Brain Show Enhanced Colony Formation Only After a Period of Culture in High Oxygen

**Figure 4:**
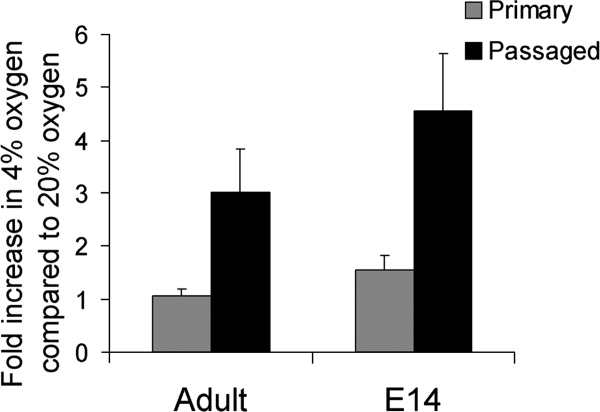
Brain-derived definitive neural stem cell (dNSC) colony numbers are increased by low-oxygen culture after exposure to normoxic conditions. Primary dNSCs from embryonic (E14) ganglionic eminence or adult lateral ventricle showed little response to low oxygen, adult t_6_ = 0.20, *p* > .05, *n* = 4, embryonic t_4_ = 5.24, *p* > .05. Passaged dNSC colonies, which had been exposed to 20% oxygen for at least 7 days, were positively influenced by low-oxygen culture. Colony number in 4% oxygen was significantly greater than in 20% oxygen: adult: t_8_ = 3.06, embryonic t_6_ = 3.68, *p* < .05 for each, *n* = 4,5. The average fold increases ± SEM in primary dNSC colony number in 4% oxygen over that in 20% oxygen are shown.

We tested whether low-oxygen culture had a similar enhancing effect on dNSC cultures from the adult or embryonic brain. In primary cultures from the adult subependymal zone or embryonic ganglionic eminence, low oxygen had little effect on the number of dNSC colonies that formed (Fig. 4). To control for the fact that ESCs have been cultured for long periods of time at 20% oxygen and tissue removed from the brain has never been exposed to high-oxygen conditions, we also tested for an effect of low-oxygen culture on dNSCs that had been previously grown in 20% oxygen. Both adult and embryonic dNSCs showed enhanced colony formation in low oxygen after one or more passages in 20% oxygen (Fig. 4).

## DISCUSSION

**Figure 5:**
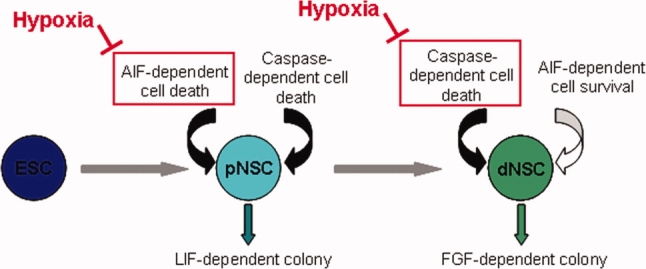
Double dissociation of cell death pathways in response to variable oxygen between pNSCs and dNSCs. Single ESCs acquire a neural identity when plated at low density in minimal media. ESCs first give rise to pNSCs, of which few survive as they undergo both caspase9-dependent and AIF-dependent cell death. pNSCs can be passaged to give rise to FGF-dependent dNSCs. dNSCs are also subject to cell death, but primarily through caspase-dependent processes. AIF has a prosurvival role in dNSCs, opposite to its actions in pNSCs. When pNSCs are derived in 4% oxygen, AIF-dependent but not caspase9-dependent cell death is attenuated and many more pNSCs survive to give rise to LIF-dependent colonies. Low-oxygen culture diminishes caspase-dependent apoptosis in dNSCs and AIF-dependent cell death is unaffected. Response to low oxygen double dissociates cell death pathways in these two early NSC populations. Abbreviations: AIF, apoptosis-inducing factor; dNSC, definitive neural stem cell; ESC, embryonic stem cell; FGF, fibroblast growth factor; LIF, leukemia inhibitory factor; pNSC, primitive neural stem cell.

pNSCs have been demonstrated to undergo a significant survival challenge when derived from ESCs in the colony-forming assay [3]. In this study, we show that cell death in pNSCs can be prevented by culture in low oxygen, and that these culture conditions do not inhibit the acquisition of a neural fate. We also find that there is substantial cell death occurring in dNSC cultures that can be similarly attenuated in low-oxygen culture. pNSCs clonally derived from ESCs are normally subject to caspase9-dependent apoptosis as well as AIF-mediated cell death. Clonal dNSCs are also subject to caspase-dependent cell death but, in contrast, require AIF for their survival. Inhibition of AIF-dependent cell death occurred when pNSCs were cultured in low-oxygen conditions, but in dNSCs, low oxygen attenuated caspase-mediated apoptosis. These findings serve to double dissociate these two cell death pathways in response to variable oxygen in these two distinct, sequential NSC populations (Fig. 5).

AIF has been suggested to be important for cell death occurring in the early embryo, as cavitation of embryoid bodies was found to be disrupted in AIF mutant ESCs [11]. However, subsequent work showed that *aif-/Y* mutant mouse embryos are not deficient in proamniotic cavity formation and are indistinguishable from wild-type embryos at early stages [19]. *aif-/Y* ESCs are resistant both to growth factor deprivation and to serum starvation, and as ESCs are deprived of serum in the neural colony-forming assay, an increase in pNSC formation in AIF mutant cells would be predicted. This is the case, however there is an even larger increase in pNSC colony formation in caspase9 mutant ESCs. The difference in cell death pathways involved in the low-oxygen effect is then not simply a consequence of acute serum starvation in pNSCs. We found that whereas caspase9 mutant pNSCs gave rise to more dNSC colonies, AIF mutant pNSCs actually gave rise to fewer clonal dNSC colonies, indicating that AIF is required for their survival. A prosurvival role for AIF is indicated by *aif-/Y* embryos, which show massive cell death after E9.0, and by studies of the Harlequin mouse, an AIF hypomorph that has increased cell death in cerebellar and retinal neurons [20].

Little is known about cell death in early NSC populations in the mammalian embryo. Cell death in the developing brain has been well studied in populations of immature, postmitotic neurons, which compete for target-derived trophic factors to be maintained. However, there is also evidence for cell death occurring in early, proliferating neural progenitors. In the rat brain, rare apoptotic cells are present in the E10 neuroepithelium, although earlier time points were not examined [21]. During later embryogenesis, cell death can be detected in BrdU-positive, proliferating cells throughout the brain [22,23]. Transgenic mice lacking key components of cell death pathways also show decreased cell death in the developing brain with resulting gross malformations and embryonic or early postnatal mortality [24–27]. In particular, *caspase9*^−/−^ mice have abnormally large brains, including an enlarged ventricular zone [25], defects consistent with altered NSC or neural progenitor cell survival. In contrast, AIF mutant mice do not show an enlarged brain. According to our model, AIF knockout mice may have an increased pNSC number, however, after the transition to dNSCs, survival would be compromised. AIF knockout embryos do not survive past E11.5 and embryos are severely reduced in size after E9.0. A reduction in the anterior brain has been reported at E9.0, when the remaining embryo is largely normal [19].

It was unexpected that enhanced pNSC and dNSC colony formation in low-oxygen culture would be independent of HIF1. HIF1 is necessary for the effects of low oxygen on mouse ESCs. Interestingly, the response to low-oxygen culture in ESCs is opposite to that seen in pNSCs and dNSCs; cell death in ESCs is increased by low oxygen and there is also a negative effect on proliferation [8]. Oxygen-responsive pathways thus differ not only between pNSCs and dNSCs, but also between ESCs and NSCs. The importance of HIF1 and its target genes in the developing nervous system is not fully understood, as HIF1α-null embryos die at E10.0--E11.0 from severe cardiovascular defects [14]. Brain-specific HIF1α deletion has been achieved using a Nestin promoter to drive expression of Cre recombinase in HIF1α floxed mice [28]. Decreased cortical neurons seen in these mice are consistent with a survival-promoting effect of HIF1, however vasculature is also abnormal, which makes cell-autonomous effects of HIF1α difficult to discern. In any case, Cre expression in these Nestin-Cre mice was not reported before E11.5 [29], thus this model may not be useful for determining effects of HIF1 in pNSCs and their transition to dNSCs.

It is important to note that culture in 4% oxygen is hypoxic only relative to standard tissue culture procedures. We found that NSCs derived from the adult or late embryonic brain did not show a large initial response to low-oxygen culture, but had greatly enhanced colony formation after a period of exposure to 20% oxygen. Indeed, NSCs in vivo are exposed to oxygen levels that are much closer to the levels experienced in our low-oxygen culture than to those in conventional, high-oxygen tissue culture. Our results indicate that cellular responses to oxygen are likely not altered when these cells are plated immediately at 4% oxygen, as there has been little absolute change in oxygen levels. However, sustained low-oxygen conditions are beneficial in culture of primary embryonic mouse cortical progenitors, which normally cannot be maintained for an extended period of time but can be continually expanded in low oxygen [30]. In this system, low oxygen acts primarily on the most undifferentiated precursor cells. Ischemia is not required in vivo to activate oxygen-responsive pathways, and expression of HIF occurs during normal development of the embryo, including within the proliferating neuroepithelium [31]. Our data suggest that both HIF-dependent and HIF-independent oxygen-responsive pathways may be activated within NSCs in the developing embryo.

## CONCLUSION

There has been little investigation into the influence of oxygen levels on early stem cell populations. In this work we demonstrate that significant cell death occurs in pNSCs and dNSCs in standard cell culture conditions and that this can be inhibited at near physiological levels of oxygen. Cell death in early neural tissue is normally low, and limited oxygen availability during embryogenesis may be one mechanism preventing apoptosis of both pNSCs and dNSCs, although these distinct populations experience different survival challenges and use the antagonism or activation of different cell death pathways to avoid cell death.

## DISCLOSURE OF POTENTIAL CONFLICTS OF INTEREST

The authors indicate no potential conflicts of interest.
