# Supplementary figures and images for: Low Oxygen Enhances Primitive and Definitive Neural Stem Cell Colony Formation by Inhibiting Distinct Cell Death Pathways
Source: Stem Cells. 2009 Aug;27(8):1879–86. doi: 10.1002/stem.96 (PMC2771103; doi:10.1002/stem.96)

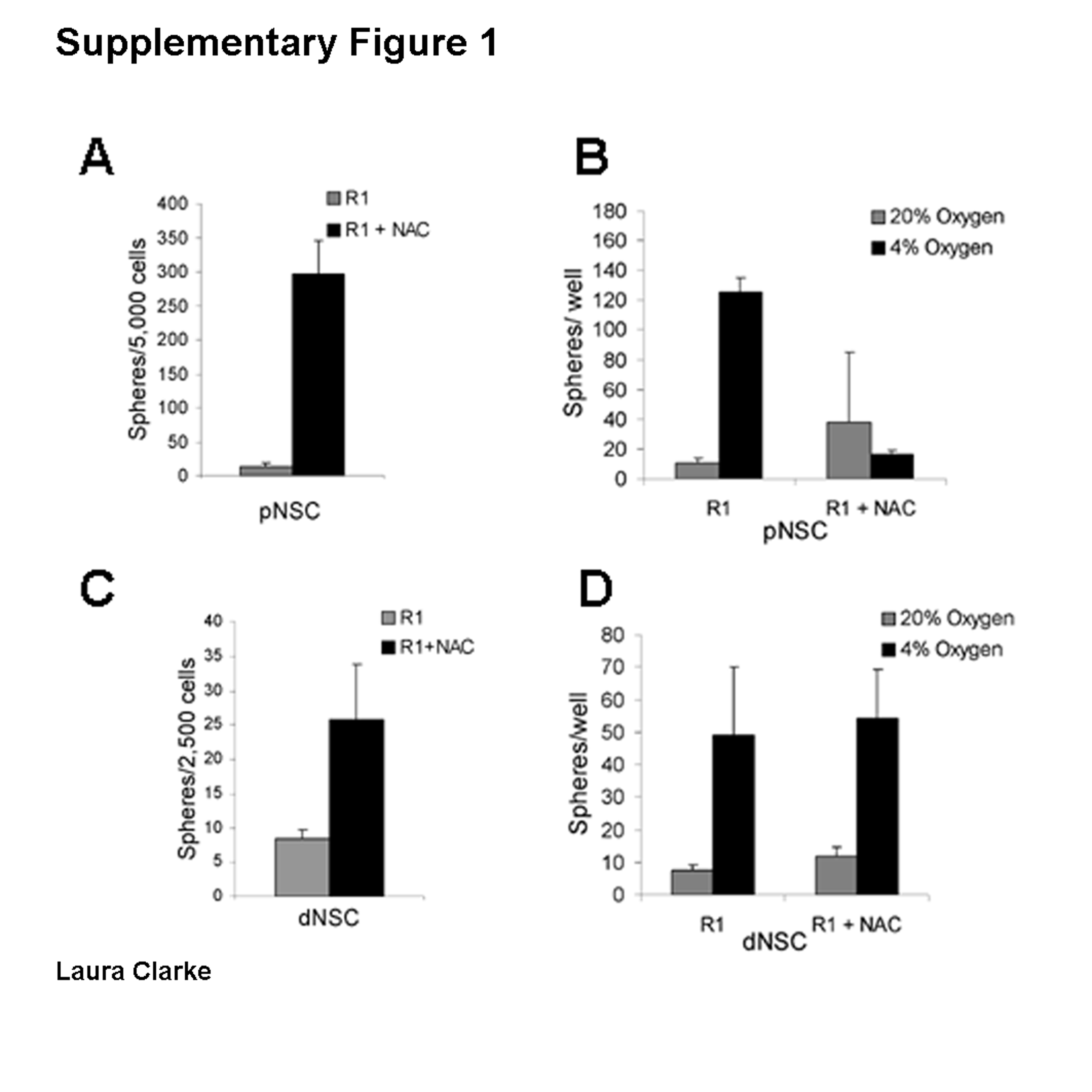

Supplement: Supplementary file 1 [file stem0027-1879-SD1.tif]

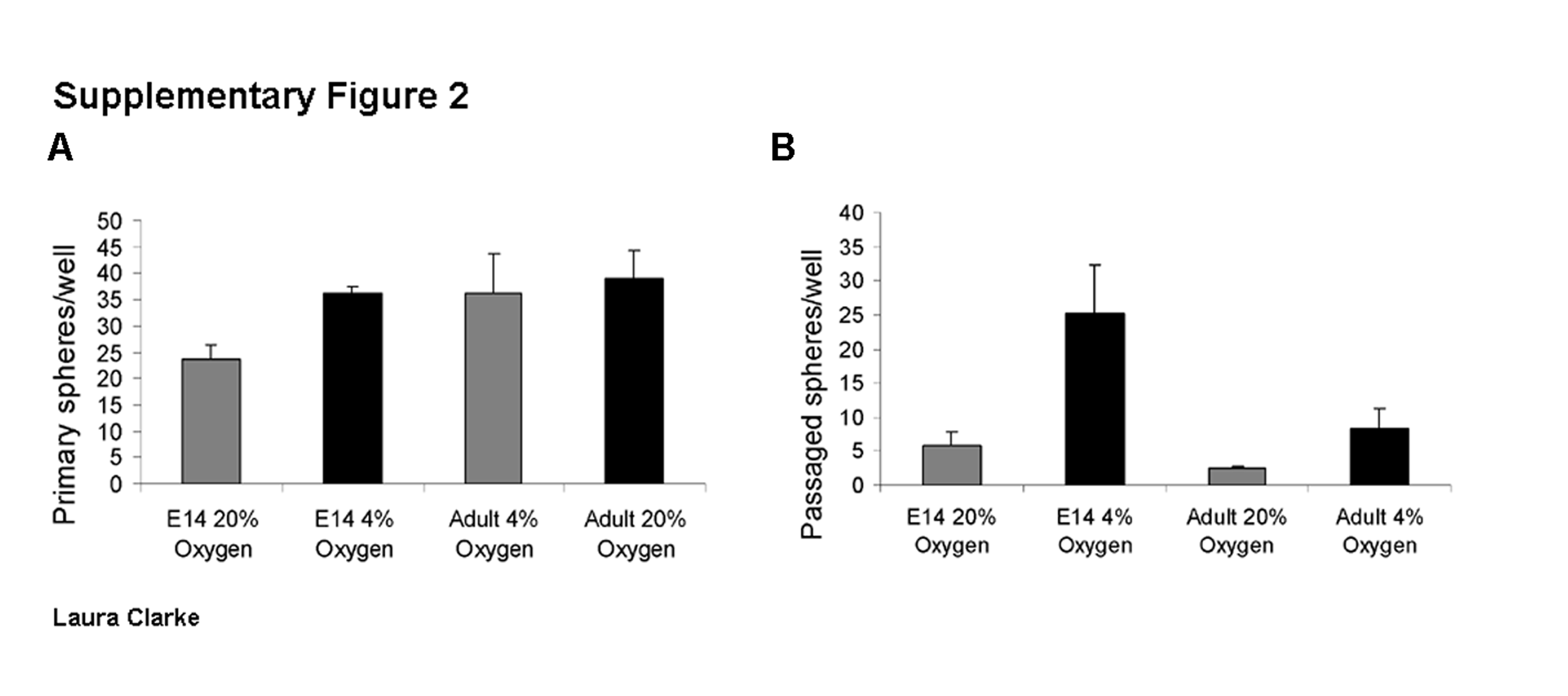

Supplement: Supplementary file 2 [file stem0027-1879-SD2.tif]
